# Comment on “Early cancer detection by serum biomolecular fingerprinting spectroscopy with machine learning”

**DOI:** 10.1038/s41377-024-01663-3

**Published:** 2025-01-20

**Authors:** Ivan A. Bratchenko, Lyudmila A. Bratchenko

**Affiliations:** Laser and Biotechnical Systems Department, Samara National Research University, Moskovskoe shosse 34, Samara, 443086 Russia

**Keywords:** Raman spectroscopy, Biophotonics

Dear Editor,

In their recent publication, Dong et al.^1^ demonstrated high performance of SERS-based liquid biopsy of human serum analysis for earlier cancer detection. Moreover, this study was highlighted in News & Vies section of Light Science and Applications journal by Shi et al.^2^. No doubt, different optical techniques nowadays are widely used for the analysis of biofluids and liquid biopsy^3–10^ and demonstrate high accuracy in possible clinical applications. At the same time, the paper by Dong et al. may be treated incorrectly due to the drawbacks in spectral data analysis.

First of all, Dong et al. proposed to evaluate provided classification models based on separation of acquired spectral data into training and test sets. This is an adequate approach widely used in machine learning to prove classification models stability^11^. In this approach it is required to demonstrate performance of the model both on the training set and on the test set (comparability of performance in test set and performance in training set proves the proposed model stability). Dong et al. divided their spectral data in 4:1 ratio (training:test), but surprisingly demonstrated only one value to characterize the proposed models. Actually Dong et al. mixed the data from training and test groups in such value. It is clearly seen on the data, presented in figures and text, that Dong et al. demonstrates performance of the proposed classification models on the whole dataset instead of two values for training and test. As an example Dong et al. collected spectra from serum of 244 lung cancer (LC) patients and 324 healthy controls (HC). The authors divided the spectral dataset as 195 LC with 259 HC for the training and 49 LC and 65 HC for the test. But the final performance of the LC vs HC classification model is demonstrated for 244 = 195 + 49 LC patients. In this regard, the actual performance of the model is hidden from readers.

For better understanding, let us assume that we try to classify 50 vs 50 samples and divide them in 4:1 (80:20 samples) ratio for training and test. If the proposed classifier is overestimated, it will provide 100% accuracy in training (correctly classifying 80 samples) and will provide a random chance of classification (50%) in test. In this case 10 out of 20 samples will be classified correctly in test. The correct representation of results for such a scenario is demonstration of 100% accuracy for training (80 samples) and 50% accuracy for test (20 samples). In this case the reader clearly sees that the performance of the proposed classifier drops significantly and such classifier may not be used in practical applications. On the other hand, if we demonstrate the accuracy of such a classifier for the whole dataset (100 samples) we will find that only 10 out of 100 samples were identified incorrectly and the accuracy of classification is 90%. The actual accuracy of classification in this hypothetical example (50%) is masked by the overestimated model predictions in the training set. Dong et al. provided accuracies from 81% to 99% for the different types of cancer classification and failed to demonstrate data separately for training and test. In this regard actual accuracy of cancer detection by Dong et al. may vary from 50% (as in the presented hypothetical scenario) to 99% and only the data from the test sets may show reliable values.

Second important point in the paper by Dong et al. is that the authors demonstrated only one possible split of spectral data. If one divides data in 4:1 ratio it is possible to divide the data in different ways (when different portions of spectral data appear in the test set). In each of such splits the achieved accuracy of spectral data classification will differ^12^ and in this regard it is important to demonstrate the resulting accuracy for multiple splits as Mean ± Deviation/Confidence interval. Only performance shown in the interval form allows one to clearly understand the capabilities of the proposed method.

Important to note, that in their highlight Shi et al. evaluated only performance values provided by Dong et al. and missed critical evaluation of machine learning approaches utilized in the novel proposed SERS-based liquid biopsy technique. Without critical evaluation numeral novel approaches demonstrate overoptimistic results for optical and liquid biopsy techniques^13–15^. Along with the high accuracy demonstration it is required to demonstrate reliability of highlighted techniques, or the readers may receive the untrustworthy information.

In conclusion, the discussed paper provides very important insight into the possibilities of SERS-based liquid biopsy combined with machine learning to analyze serum in order to detect cancer. However, in order to confirm that the obtained high performance values are correct, the authors should estimate the correctness of machine learning approaches proposed to analyze spectral data.
